# Optimal phenology of life history events in *Calanus finmarchicus*: exit from diapause in relation to interannual variation in spring bloom timing and predation

**DOI:** 10.1093/plankt/fbae028

**Published:** 2024-06-07

**Authors:** Thomas R Anderson, Dag O Hessen, Wendy C Gentleman, Andrew Yool, Daniel J Mayor

**Affiliations:** Marine Systems Modelling, National Oceanography Centre, Southampton SO14 3ZH, UK; Centre of Biogeochemistry in the Anthropocene, Department of Bioscience, University of Oslo, Oslo 0316, Norway; Department of Engineering Mathematics, Dalhousie University, Halifax, NS, B3J 1B6, Canada; Marine Systems Modelling, National Oceanography Centre, Southampton SO14 3ZH, UK; Department of Biosciences, Hatherly Building, University of Exeter, Exeter EX4 4PS, UK

**Keywords:** seasonal lipid pump, copepod, Calanus finmarchicus, diapause exit, trade-offs, trait optimization, Norwegian Sea, phenotypic variance

## Abstract

Respiration of lipids by copepods during diapause (overwintering dormancy) contributes to ocean carbon sequestration via the seasonal lipid pump (SLP). Parameterizing this flux in predictive models requires a mechanistic understanding of how life history adaptation in copepods shapes their timing of exit from diapause. We investigate the optimal phenology of *Calanus finmarchicus* in the Norwegian Sea using an individual-based model in which diapause exit is represented as a trait characterized by phenotypic mean and variance. Without interannual variability, optimal exit correlated with the onset of the spring phytoplankton bloom and phenotypic variance was of no benefit. In contrast, copepods endured reduced fitness and adopted bet-hedging strategies when exposed to interannual variability in bloom timing and predation: later exit from diapause and phenotypic variance maintained adult numbers in anomalous late-bloom years. Exit nevertheless remained well before the peak of the bloom which is a favorable strategy when low predation early in the year enhances survival of eggs and early developmental stages. Our work highlights the complex interactions between *C. finmarchicus* and its environment and the need for improved understanding of bet-hedging strategies and the cues of diapause exit to progress the representation of the SLP in global biogeochemical models.

## INTRODUCTION

Calanoid copepods are integral to the structure and function of high-latitude marine ecosystems and associated biogeochemical cycling of carbon (C) and nutrients. The species *Calanus finmarchicus*, which occurs throughout the North Atlantic (Planque and Batten, 2000), acts as an important trophic link between primary producers and higher trophic levels such as fish, seabirds and whales (Bachiller *et al*., 2016; Plourde *et al*., 2019), and its fecal pellets contribute significantly to the export flux of C from the surface to deep ocean (Bathmann *et al*., 1987). This species enters a metabolically quiescent period termed diapause towards the end of the productive season in which animals migrate into deep waters that provide a refuge from visual predators (Kaartvedt, 1996; Dale *et al*., 1999). The lowered rates of metabolism during this period of dormancy are sustained by respiring lipid reserves that are accumulated during the active phase as late-stage copepodites (Jónasdóttir *et al*., 2019). The respired C thereby released in deep waters contributes to ocean C sequestration via the “Seasonal Lipid Pump” (SLP) and may be similar in magnitude to that due to sinking particles at high latitudes (Jónasdóttir *et al*., 2015; Visser *et al*., 2017; Anderson *et al*., 2022; Tarling *et al*., 2022).

The SLP is not represented in the current generation of ocean biogeochemical models that are embedded within the Earth System Models used to project the response of marine ecosystems and ocean C sequestration to changing climate. An important prerequisite is a mechanistic understanding of the drivers of life history events in copepods, including diapause. Descent into deep water at the onset of diapause occurs after the accumulation of lipid reserves in spring and summer (Miller *et al*., 1998a; Rey-Rassat *et al*., 2002; Pond *et al*., 2012). After overwintering, exit from diapause in *C. finmarchicus* coincides with the spring phytoplankton bloom that provides food resources to fuel egg production, growth and development (Niehoff *et al*., 1999; Heath *et al*., 2000a; Hirche *et al*., 2001; Broms *et al*., 2009). The optimal timing of diapause exit involves a trade-off between maximizing opportunities for growth and reproduction in surface waters versus surviving adverse periods of low food availability and avoiding predation (Dahms, 1995; Pierson *et al*., 2013; Varpe and Ejsmond, 2018; Visser *et al*., 2020). This trade-off is complicated by environmental stochasticity (Fiksen, 2000; Bandara *et al*., 2021a). *C. finmarchicus* experiences interannual and geographical variation in environmental drivers including between-year timing of the spring phytoplankton bloom (Henson *et al*., 2006, 2009; Platt *et al*., 2010). The means by which copepods optimize diapause exit when faced with this variation is not well understood, all the more so because of a lack of consensus on the associated cues which may include light (photoperiod), food availability, predation, exhaustion of lipid reserves and internal biological clocks (Hirche, 1996; Johnson *et al*., 2008; Bandara *et al*., 2018, 2021b and references therein).

Optimality-based modeling, where functional diversity within a population is represented by key traits that vary among individuals, provides a means of investigating life history trade-offs in cyclical and fluctuating environments (Smith *et al*., 2011; Varpe, 2017). Previous modeling studies have shown that *C. finmarchicus* may use bet-hedging (risk-spreading) strategies to cope with long-term variability in phytoplankton blooms and predation, where short-term fitness is sacrificed to promote long term population survival (Poethke *et al*., 2016; Varpe, 2017). One way of hedging bets is for a population to exit diapause on a range of dates although many individuals may incur reduced fitness as a consequence (Huse *et al*., 2018). Using exhaustion of energy (lipid) reserves as the cue, the model of Bandara *et al*. (2021a) generated a year-round continuum of *C. finmarchicus* diapause exit dates in an idealized environment with seasonal cycles of environmental heterogeneity and stochastic variation over 100 years. While providing flexibility this strategy was, however, wasteful as many adults emerging from diapause likely faced food deprivation and starvation. Using a similar model but with “wake-up-day” from diapause cued by daylength, Fiksen (2000) found that optimal exit in *C. finmarchicus* is delayed by environmental stochasticity.

Our aim here is to investigate the bottom-up and top-down evolutionary drivers that influence the timing of diapause exit in the high-latitude copepod *C. finmarchicus* when faced with interannual variability in bloom timing and predation risk. Understanding these drivers paves the way for the development of generic parameterizations of diapause and the SLP for use in global ocean biogeochemical models. We use a new individual-based stoichiometric model of *C. finmarchicus* that divides the life cycle of into six phases, has separate state variables for structural and lipid biomass and which propagates over multiple generations. The model has three unique aspects compared to previous optimality studies: (i) growth, development and egg production are simulated using a state-of-the-art stoichiometric equations that include physiologically based metabolic terms for both C and nitrogen (N) that are measurable by observation and experiment (Anderson *et al*., 2022; Mayor *et al*., 2022); (ii) trait convergence occurs spontaneously via a process that mimics natural selection (Follows *et al*., 2007) without the use of optimization algorithms; (iii) a single trait is represented, the day of year which copepods exit from diapause, but which has two properties, optimum (mean) and phenotypic variance, both of which are optimized in the model simulations. Phenotypic variance incorporates genetic and environmental factors (Willmore *et al*., 2007; Schou *et al*., 2020), including phenotypic plasticity, which is the expression of multiple phenotypes in response to environmental conditions. Phenotypic variance and plasticity are both under genetic control and thereby subject to evolutionary forces (Via *et al*., 1995; Liefting and Ellers, 2008; Bruijning *et al*., 2020; Lai and Schlötterer, 2022). In this context, it is impossible to separate between epigenetic factors and change in gene frequency, alleles, mutations or other “hard wired” genetic drivers, but this has no bearing on the model predictions. Optimal values of the mean and variance, i.e. those that confer the greatest fitness in terms of fecundity and survivorship, are propagated from one generation to the next with greatest frequency as each model simulation progresses, eventually converging on a single unique value for each. The environmental forcing in our study is based on Station Mike in the Norwegian Sea (66°N, 2°E), using a 10-year sequence for spring bloom timing and temperature from which predation pressure is calculated using an exponential relationship. Results are discussed in context of the need to represent copepod phenology in biogeochemical models of the ocean C cycle.

## METHODS

The *C. finmarchicus* modeling undertaken herein has as its foundation the LILICOP_1.0 model that simulates the growth, development and reproduction of an individual copepod throughout its life cycle (Anderson *et al*., 2022). LILICOP has C and N as currencies, explicitly separates structural biomass and lipid storage as state variables and incorporates the latest stoichiometric theory in which metabolism includes explicit terms for biomass turnover, other basal costs and specific dynamic action (Anderson *et al*., 2020, 2021). As well as providing physiological realism, an advantage of metabolic stoichiometry is that parameter values for these processes are available directly from observations and experiments (Mayor *et al*., 2022). A brief outline of LILICOP_1.0 is provided below, followed by a description of the new trait-based version of the model, LILICOP_2.0, including the formulations of predation mortality and phenotypic variance as well as the 10-year forcing for Station Mike.

### Individual copepod model

A full description of LILICOP_1.0, including listings of functional dependencies, parameter values and justification thereof, is provided in Anderson *et al*. (2022). The parameter values used here are unchanged from their original settings, unless otherwise stated. The life cycle of *C. finmarchicus* is divided into six phases that are intimately linked to the accumulation and use of reserve lipids (Anderson *et al*., 2022). Non-feeding stages (eggs and nauplii NI and NII) collectively comprise phase 1 after which copepods start feeding, fueling growth (phase 2; NIII to copepodite CII) and both growth and accumulation of reserve lipid (phase 3; development to CV). Animals then cease feeding and enter diapause which takes place in deep waters where predation losses are minimal (phase 4), followed by gonad development (phase 5), which also occurs in deep water without access to food. Surviving adults re-emerge at the surface, ready to produce eggs fueled by food intake (phase 6). The model does not include capital production in which eggs are produced using maternal lipid reserves (Varpe *et al*., 2009). The number of eggs produced this way may not be large because adult females have limited lipid reserves when exiting from diapause (Anderson *et al*., 2022).

Development during phase 1 is temperature dependent, taking 7 days at 10°C. The transitions between phases 2 to 3, and 3 to 4, occur when critical biomasses are reached. In case of the latter, which is the point at which *C. finmarchicus* enters diapause, Anderson *et al*. (2022) used an individual biomass of 15.9 μmol C, comprising 6.5 μmol C structure and 9.4 μmol C as lipid. The lipid reserve is a critical parameter for diapause (Anderson *et al*., 2022) and preliminary work with the current model was undertaken to optimize it alongside phenological traits. In nearly all cases it settled on a value close to the original (80%), giving lipid biomass of 7.52 μmol C and total individual biomass 14.0 μmol C; these values are used in the simulations described herein. Phase 5 lasts 14 days and is not strictly part of diapause. We nevertheless denote the day of year that mature females (CVI) return to surface waters as the “diapause exit date” because diapause exit and re-emergence are generally thought of as synonymous. Diapause exit is a model parameter in LILICOP, without invoking a specific mechanism or cue. Calculated metabolic rates are temperature-dependent (Q_10_ of 2), with default values that are two orders of magnitude lower in diapause. Grazing on diatoms, non-diatoms, microzooplankton and detritus (model forcing; see below) fuels development (growth and lipid accumulation; phases 2 and 3) and egg production (phase 6). It is calculated using a multiple-resource Sigmoidal (Holling III) functional response with a temperature-dependent maximum rate (Q_10_ of 2). There is no feeding during phases 4 and 5. If an individual avoids being consumed by predators, it ultimately starves and dies when food runs out (the simulated life cycle lasts no more than two years).

There is no explicit representation of the physical water column. Rather, animals reside in either surface or deep waters at any one time, with instantaneous transition between the two assumed for simplicity. Environmental forcing for surface waters is off-line using food fields (diatoms, non-diatoms, microzooplankton and detritus) and temperature taken from Station Mike’s location as simulated by the NEMO-MEDUSA global biogeochemical model, at 5-day resolution (Yool *et al*., 2013).

### Trait-based population model

**Fig. 1:**
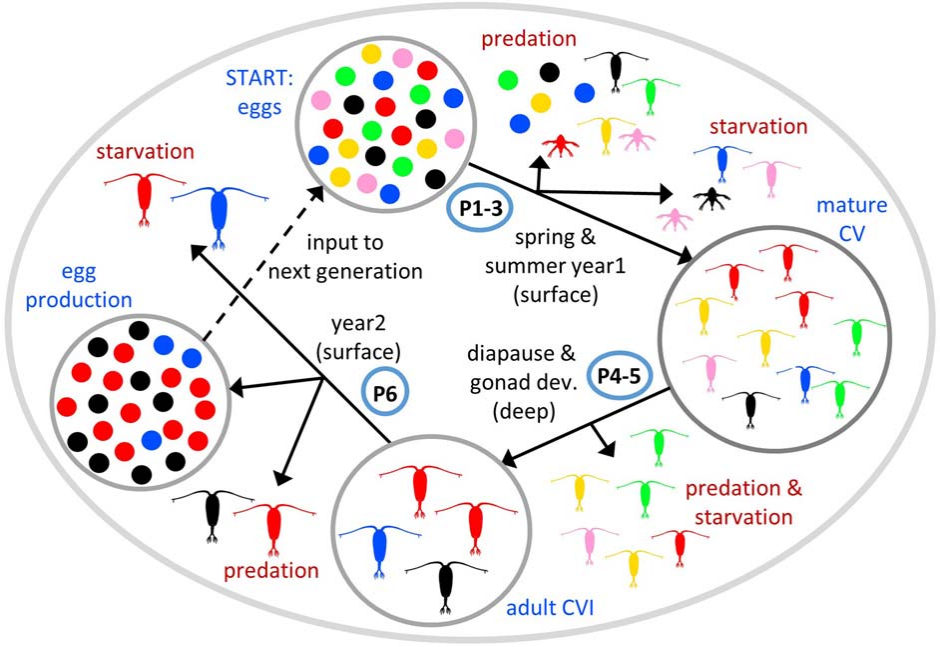
Schematic illustrating survival and reproduction of *Calanus finmarchicus* individuals throughout their life cycle in the LILICOP_2.0 model, for a single generation of sub-population size 24. Different colors represent variation in the value of a single trait within the population; change in frequency of these colors indicates selection over time. Eggs produced during the second year are used as input to repeat the cycle over multiple generations (dashed line). P1–6 are phases 1–6 as described in the text.

LILICOP_2.0 enhances the original LILICOP_1.0 model in four ways: (i) it simulates a sub-population (representative sample of the population as a whole) of *C. finmarchicus* that propagates over multiple generations, (ii) diapause exit day of year is incorporated as a trait with two properties, mean and phenotypic variance, that vary among individual copepods, (iii) optimal values of these properties emerge over multiple generations via a process that mimics natural selection (Follows *et al*., 2007) and (iv) mortality due to predation is introduced as a stochastic loss term that is imposed throughout the copepod life cycle. The growth, development, reproduction and fate of each individual copepod is followed in turn as it transitions through the six phases of the life cycle (Fig. 1). Losses of individuals occur due to starvation and predation. Traits are inherited unchanged as they pass from adult females to their progeny. On completion of the first generation a fixed number of eggs, n_pop_ (sub-population size), is passed forward as the starting point for the next generation, ensuring that the predicted frequency distribution of trait values and egg spawn dates in the total eggs produced is maintained. Natural selection in the face of starvation and predation pressure will favor traits that enable survival and maximize fecundity, e.g. as illustrated by the propagation of red, blue and black eggs in Figure 1. Trait convergence was reached in all the simulations that we conducted; i.e. all individuals within the sub-population ended up with the same values for trait mean and variance. It is not a given that trait convergence necessarily occurs; the fact that it uniformly did so indicates that selective pressures relating to maximizing fitness in individual copepods are strong, at least for the environmental forcing imposed in this study.

Sixteen values of trait mean are used to represent the potential range of optimal diapause exit date within the population, starting at Julian day 50 and 10 days apart, i.e. days 50, 60, 70, …, 200, along with five values of phenotypic variance (normally distributed) specified in terms of standard deviation: SD = 0 (no variance), 10, 20, 30 and 40 days (see below). The total number of possible trait combinations is 16 × 5 = 80. Egg spawn date is assigned 51 bins 5 days apart and starting at 50, giving dates of 50, 55, 60, …, 300 (the growing season; eggs spawned out of this range are non-viable because of food scarcity over winter; Anderson *et al*., 2022). The total number of possible combinations of traits and egg spawn dates is 80 × 51 = 4080. A large sub-population size is used at initialization to ensure that the stochastic parameterization of predation mortality does not unduly influence the predicted trait values at convergence; we used n_pop_ = 408,000, providing 100 individuals for each trait combination that are spread across the range of egg spawn dates, except for sensitivity analysis using high predator mortality when we used n_pop_ = 2,040,000. These values of n_pop_ are maintained for the first 20 generations by which time the trait distribution had been significantly reduced by natural selection. Sub-population size was thereafter decreased to n_pop_ = 100,000, saving on run time.

The LILICOP_2.0 model is coded in R and runs stand-alone on a standard PC. Running an on-line version of the model, i.e. in which individual copepods are simulated using the full suite of differential equations, is not feasible because of the prohibitive run-time associated with simulating a large number of individuals over many generations. We solved this problem by developing LILICOP_2.0 as an off-line model in which copepod growth and development are read in from look-up files generated beforehand by running LILICOP_1.0 for all possible combinations of diapause exit date and egg spawn date. The progression of an individual copepod through its life cycle is then determined by stepping through the relevant off-line file where development stage, size of structural and lipid biomass, egg production and starvation mortality are recorded on a daily basis.

### Predation mortality

There is no explicit representation of predator biomass and consumption rates in the model. Instead, predation is calculated as a stochastic process: a random number between 0 and 1 is generated at each daily time step during the simulation of an individual copepod and if it is below a specified probability of predation mortality (*m*) the animal dies. Mortality due to predation is thought to increase during the growing season as the marine ecosystem ramps up over time. This increase can be modeled by using temperature as a proxy for predation pressure where both predator abundance and consumption rates increase during the warmer months (Speirs *et al*., 2006; Neuheimer *et al*., 2010; Banas *et al*., 2016; Maud *et al*., 2018; Aarflot *et al*., 2022). The exponential relationship of Plourde *et al*. (2009) was used by Neuheimer *et al*. (2010) to describe temperature-dependent mortality of copepods due to predation pressure for different regions off the east coast of Canada, *m*(*T*) = *a* exp(*bT*), which can be reformulated as:


(1) \begin{equation*} m(T)=m\left({T}_{\mathrm{ref}}\right)\exp \left(0.214\left(T-{T}_{\mathrm{ref}}\right)\right) \end{equation*}

where *m*(*T*) and *m*(*T*_ref_) are mortality rates at temperatures *T* and reference *T*_ref_, respectively; *a* = *m*(*T*_ref_)exp(−*bT*_ref_) and b = 0.214. We use this relationship, which equates to a Q_10_ of 8.5, in conjunction with *T*_ref_ = 9°C, which is the average surface temperature throughout the 10-year interannual sequence during the growing season at station Mike (days 100–300).

Predation mortality is assumed to vary with development stage in common with other models and field estimates (Lynch *et al*., 1998; Ohman *et al*., 2004; Moll and Stegert, 2007; Maps *et al*., 2010 ; Alver *et al*., 2016). Parameters *m*_1_, *m*_2_ and *m*_3_ define daily probabilities during development (phases 1–3), diapause and gonad development (phases 4–5) and for adults (phase 6), respectively; *m*_1_ and *m*_3_ covary with temperature (Eq. 1), i.e. when copepods are in surface waters. Typical mortality rates for copepodites and adult female *C. finmarchicus* used in previous studies are 0.02–0.05 d^−1^(Lynch *et al*., 1998; Pershing *et al*., 2009; Maps *et al*., 2010). Assuming that these rates are dominated by predation (Davis, 1984; Sell *et al*., 2001), we use daily probabilities of *m*_1_ = 0.03 d^−1^ for pre-diapause individuals and *m*_3_ = 0.02 d^−1^ for post-diapause adults, with *T*_ref_ = 9°C. Higher rates can occur in eggs and nauplii but these may be due to non-predatory losses that are represented separately in the model. The rate of predation in phases 4 and 5 is set an order of magnitude lower, at 0.002 d^−1^ (Maps *et al*., 2010; Alver *et al*., 2016) given that dark deep waters provide refuge from visual predators.

### Phenotypic variance

**Fig. 2:**
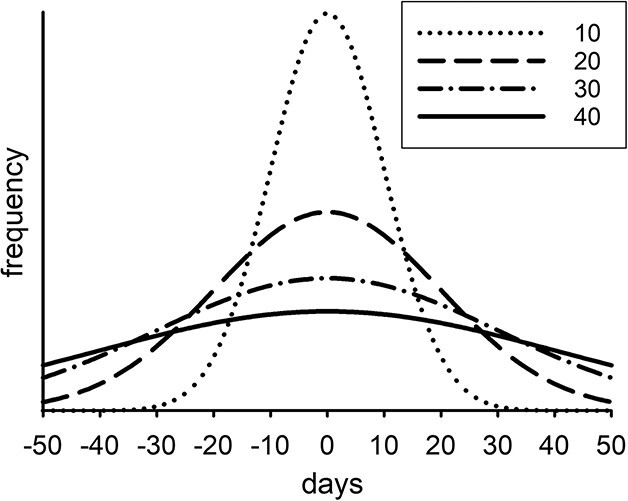
Frequency distributions for phenotypic variance of diapause exit with standard deviation, SDPV = 10, 20, 30, 40 days.

Previous optimality modeling studies have included genetic diversity (Fiksen, 2000; Bandara *et al*., 2021a). Variability in morphology, physiology and behavior can also derive from phenotypic diversity. Phenotypic variance (PV) associated with diapause exit date is represented in our model using the Gaussian distributions shown in Figure 2 with standard deviations, SDPV = 0 (no variance), 10, 20, 30 and 40 days (see Supplementary Appendix 7 for further details). Apart from zero, these distributions all represent relatively high variance at different levels. The chosen breadths of these distributions (Fig. 2) are arbitrary but nevertheless suffice to illustrate the desired effects given that intermediate values of SDPV emerged in many of the simulations. Note that the off-line scheme used in the model means that the application of PV is restricted to diapause exit dates ten days apart (60, 70, 80, …). The realized day of diapause exit for an individual copepod is its inherited mean value ± a random point within the relevant (inherited SDPV) Gaussian distribution, allocated to the nearest 10-day setting.

### Forcing

**Fig. 3:**
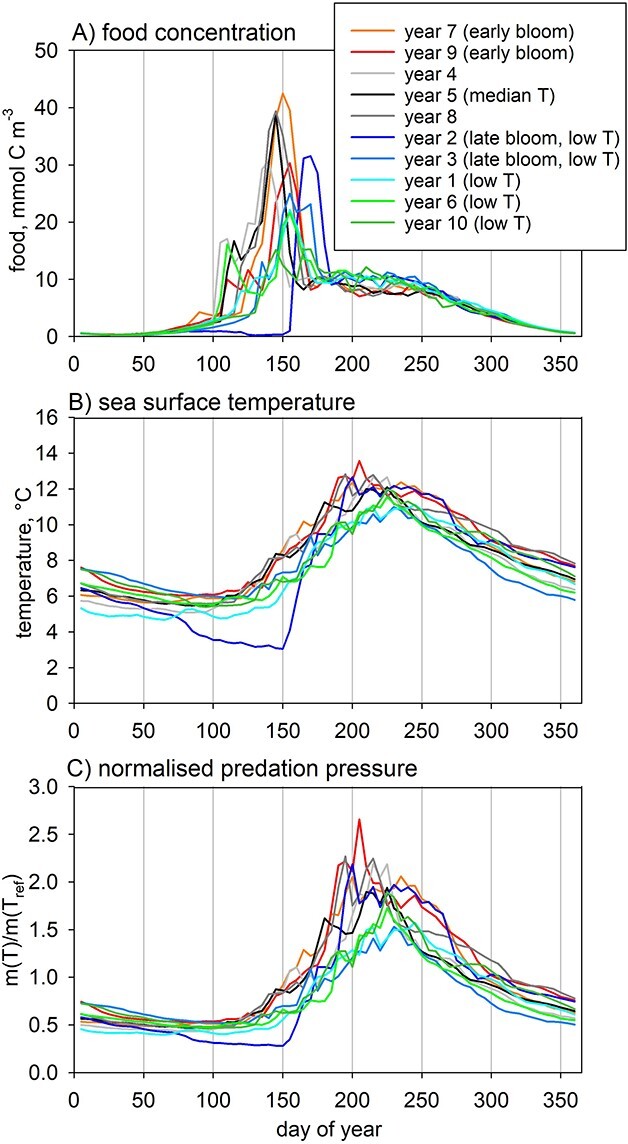
Model forcing: (**A**) food (mmol C m^−3^) and (**B**) sea surface temperature (°C), plus (**C**) normalized predation pressure (Eq. 1). The color scheme highlights different years according to bloom timing and temperature (T): early bloom (years 7, 9; orange, red), late bloom (years 2, 3; blue, pale blue), low-temperature (years 1, 2, 3, 6, 10; cyan, blue, pale blue, green, olive).

Station Mike (66°N, 2°E) is highly seasonal, characterized by a marked spring chlorophyll bloom. We simulate a 10-year interannual sequence (2000–2009) that provides a representative “present day” decade; these years will henceforth be referred to as years 1 to 10 for ease of presentation and analysis. Seasonal cycles of food fields (diatoms, non-diatoms, microzooplankton and detritus) and sea surface temperature (mixed layer averages) are taken from a high-resolution simulation of the NEMO-MEDUSA model (Fig. 3A; Yool *et al*., 2013, 2015). The timing of the spring phytoplankton bloom shows significant interannual variation, with peak food concentrations occurring earliest in year 4 (day 140) and the latest in year 2 (day 170). The seasonal trends in temperature are similar for all years, except year 2 (Fig. 3B), which has much colder temperatures in spring and a marked delay in onset of the bloom. The resulting predation pressure (Eq. 1) shows a 5-fold variation, increasing sharply in spring in tandem with temperature (Fig. 3C). A constant temperature of 4°C is used for deep-water temperature (Anderson *et al*., 2022). These forcings were used as input for the LILICOP_1.0 simulations that generated the off-line files read in by LILICOP_2.0.

## RESULTS

**Fig. 4:**
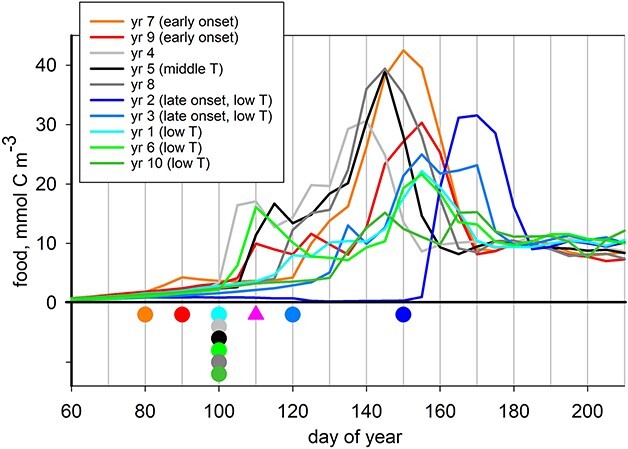
Predicted distributions of optimal exit date from diapause for the repeat-year simulations (spots) and the interannual simulation (pink triangle), plotted against seasonal cycles of food availability. The color scheme highlights different years according to bloom timing and temperature (T): early bloom (years 7, 9; orange, red), late bloom (years 2, 3; blue, pale blue), low-temperature (years 1, 2, 3, 6, 10; cyan, blue, pale blue, green, olive).

A summary of all the simulations undertaken, along with examples showing trait convergence over time, is presented in Supplementary Appendices 1 and 2, respectively. Simulations were first carried out using forcing for individual years repeated end-on-end, thereby excluding interannual variability (repeat-year, RY simulations), using ensembles of size five (fifty simulations in all). Predicted optimal exit from diapause and associated phenotypic variance converged on the same values for each year in the ensembles. Exit day varied by more than two months for the different individual years (Fig. 4), from day 80 (year 7) to day 150 (year 2), while predicted optimal phenotypic variance was zero in all cases indicating that it is of no benefit to the copepods when environmental forcing is predictable. This variation in timing of diapause exit correlates with the point in time at which food concentration first reaches 2 mmol C m^−3^ (close to 1:1; Supplementary Appendix 4, Fig. S4–1); for the purpose of interpretation we define this point as the early onset of the spring bloom and the different years are color-coded accordingly (early onset, late onset). In contrast, there was only a weak relationship with temperature (Supplementary Appendix 4).

**Fig. 5:**
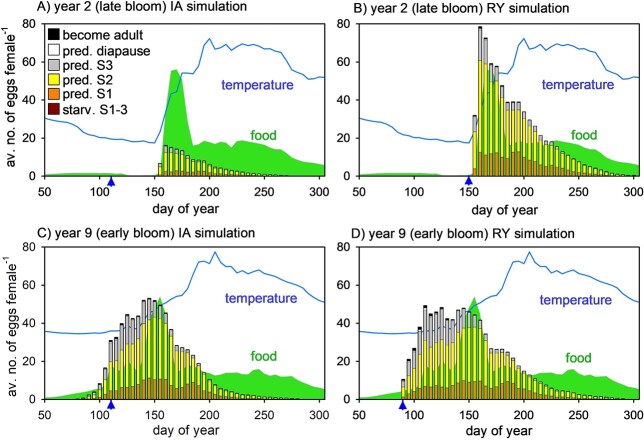
Predicted seasonal progression of average egg production per adult female in year 2 (late bloom) IA and RY simulation (panels **A** and **B**) and year 9 (early bloom) IA and RY simulations (panels **C** and **D**; blue triangles indicate optimal exit date from diapause). Bar coloration shows the fate of eggs spawned on different dates: starvation during development (phases 1–3), predation phase 1 (eggs and non-feeding nauplii), predation phase 2 (feeding and growth without lipid deposition), predation phase 3 (feeding and growth with lipid deposition), predation during diapause, and survivorship through to adults. Seasonal cycles of food (green shading) and temperature (blue line) are also shown (scaling as in Fig. 3).

A 10-member ensemble of simulations was next carried out using the full 10-year interannual sequence for Station Mike (2000–2009) repeated end-on-end (IA simulations). Trait convergence usually took at least 150 generations (Supplementary Appendix 2), after which an extra ten years were simulated to complete a full pass of the sequence for analysis purposes. Again, convergence showed 100% reproducibility, with only small differences in metrics such as adult survivorship and fecundity between different runs in the ensemble (Supplementary Appendix 3). Predicted optimal exit occurred on day 110 with phenotypic variance SDPV = 20 days noting that the latter is an intermediate value within the range investigated (10–40). Exit on day 110 is a compromise, differing from all of the individual-year optima of the RY simulations (Fig. 4). The seasonal patterns of egg production arising from the mismatch between food availability and egg production are shown in Figure 5 for years 2 and 9 (late and early bloom onset years, respectively) for the first simulation within the ensemble (results for all years are presented in Supplementary Appendix 5). Of the 2300 adults exiting diapause on day 120 in year 2 of the IA simulation, a remarkable 57% died of starvation before day 160, i.e. prior to sufficient food becoming available. Furthermore, those adults which avoided starvation were often in poor condition (reduced biomass). The resulting average fecundity per female was only 120 eggs in the IA simulation (Fig. 5A), much lower than the 695 eggs that is predicted for the corresponding RY simulation in which diapause exit takes place on day 150 (Fig. 5B). In contrast, exiting diapause somewhat too late, thereby missing the onset of the bloom, had less impact on predicted egg production. In the case of year 9, the predicted fecundities are 721 (exit date 120) and 868 (exit date 90) in the IA and RY simulations, respectively (Fig. 5C and D).

**Fig. 6:**
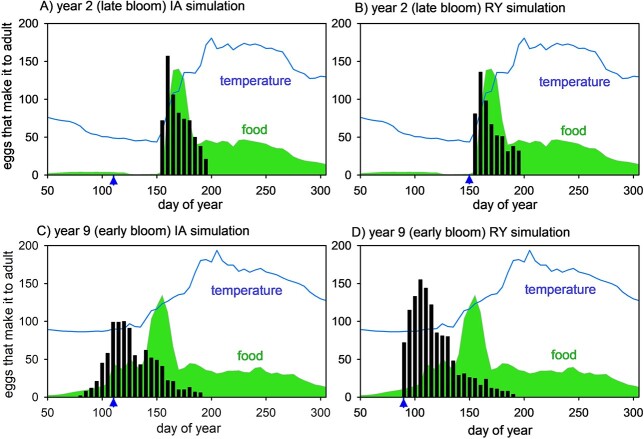
Predicted seasonal progression of total numbers of eggs that successfully develop to phase 6 (adults), ordered by egg spawn date, in year 2 (late bloom) IA and RY simulations (panels **A** and **B**) and year 9 (early bloom) IA and RY simulations (panels **C** and **D**; blue triangles indicate optimal exit date from diapause). Seasonal cycles of food (green shading) and temperature (blue line) are also shown (scaling as in Fig. 3).

Heavy predation losses (orange, yellow and gray bars in Fig. 5) mean that only a small fraction of eggs successfully make it as adults (black bars in Fig. 5). Given that predation is lowest early in the year, it is the early-spawned eggs that make the greatest contribution to successful propagation of the population as they are subject to the lowest predation losses as they develop through naupliar and copepodite stages. This is illustrated for years 2 and 9 in Figure 6, which shows the total number of eggs that successfully develop and become adults, i.e. the black bars in Figure 5 isolated and expanded, without normalization to female numbers: note the seasonal asymmetry between the bar height distributions in Figures 5 and 6. The same trend is seen in all years (Supplementary Appendix 5), although to a lesser extent in year 2. Further analysis of the success with which the sub-population is propagated from one generation to the next, which depends on the product of fecundity (eggs adult^−1^) and survivorship (adults egg^−1^), is presented in in Supplementary Appendix 6.

**Fig. 7:**
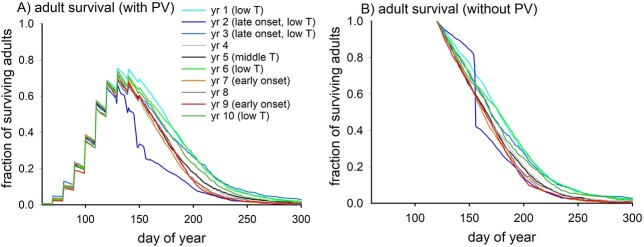
Number of surviving adults post-diapause for different years in IA simulations, normalized to the total post-diapause population: (**A**) with (standard) and (**B**) without (sensitivity analysis: see below) optimization of phenotypic variance.

The seasonal progression of adult female numbers is shown for the different years in the IA simulation in Figure 7A, normalized to the total number of adults arriving post-diapause. The saw-tooth pattern occurs because the off-line scheme used in the model only allows for 10 day intervals in diapause exit whereas attrition of numbers via mortality is calculated on a daily basis. The anomalous late-blooming year 2 stands apart from other years because of starvation losses (57%) between days 100 and 160, whereas starvation accounts for only 0.2–2.1% of adult mortality in other years. Predicted adult mortality rates were higher in warmer years because predator activity is parameterized as an increasing function of temperature (Eq. 1). The impact on fecundity was, however, minimal. Excluding years 2 (anomalous) and 5 (median temperature), an average of 829 eggs per female in the colder years (1, 3, 6, 10; lower predation pressure) is only 6.4% higher than 779 eggs of warmer years (4, 7, 8, 9; higher predation pressure).

The significance of phenotypic variance was investigated by carrying out a further 10 IA simulations with it switched off (SDPV = 0). Predicted optimal diapause exit occurred on day 120, ten days later than the IA runs with the variance included. Without the extra flexibility, the animals exited diapause later to avoid population wipeout by starvation in year 2 although high starvation losses (40%) were again incurred between days 155 and 160 of that year (Fig. 7B). With or without phenotypic variance, the copepods adopt bet-hedging strategies to allow the population to scrape by in year 2 while maximizing fitness (fecundity and survival) in “ordinary” years.

Simulations were carried out to examine the sensitivity of diapause exit to changes in predation pressure. The biggest effect was seen when the seasonal trend in predation was replaced with fixed values of *m*_1_ = 0.03 d^−1^ and *m*_3_ = 0.02 d^−1^ (rates for development to mature CV and adults, respectively), meaning that mortality is relatively higher early in the year and lower later. Predicted optimal diapause exit in the IA simulations increased from day 110 to 130 as adult copepods sought to avoid early predation losses. A similar effect was seen when re-running the RY simulations with this change in parameterization: predicted optimal exit increased in all years except year 4, with an average increase of 17 days and a maximum of 40 days in year 7. In contrast, predicted optimal exit from diapause was insensitive to increasing predation pressure by 50 or 100% while maintaining the seasonal trend, in both the IA and RY simulations (see Supplementary Appendix 1 for details of the sensitivity analysis results).

## DISCUSSION

Optimal adaptation is hard to achieve in animals that inhabit environments characterized by unpredictable fluctuations in resources (Levins, 1968; de Villemereuil *et al*., 2020). *C. finmarchicus* is exposed to significant interannual variability in the timing of the spring phytoplankton bloom (Henson *et al*., 2006, 2009; Platt *et al*., 2010). We first carried out model simulations for Station Mike in which this variability was excluded as a theoretical exercise (repeat-year, RY, simulations with forcing for individual years repeated end-on-end). The predicted optimal day of year for exit from diapause was correlated with the early onset of the spring bloom which is unsurprising because food resources fuel egg production, growth and development in copepods (Niehoff *et al*., 1999; Heath *et al*., 2000a; Hirche *et al*., 2001; Broms *et al*., 2009). Exiting early from diapause is thought to be the preferred strategy of *C. finmarchicus* (Niehoff *et al*., 1999; Head *et al*., 2000; Kaartvedt, 2000; Hirche *et al*., 2001; Stenevik *et al*., 2007) and is theoretically favorable because early-spawned eggs can make a disproportionately high contribution to overall population recruitment (Varpe *et al*., 2007). Predicted optimal phenotypic variance (PV) was zero in the RY simulations indicating that it confers little or no fitness benefit for copepods inhabiting predictable (stable) environments. Lowest phenotypic variance and plasticity are expected in animals that are subject to relatively low environmental heterogeneity in time and space (Ellers and van Alphen, 1997; Hassall *et al*., 2005; Coquillard *et al*., 2012; Schou *et al*., 2020).

The modeled copepods had to compromise and endure reduced fitness in simulations when they were exposed to a 10-year sequence of environmental forcing which includes interannual variability in spring bloom timing and predation (IA simulations). Successful propagation of the population was aided by bet-hedging (BH) strategies in which optimal fitness for any specific condition is sacrificed to promote long-term population survival under fluctuating conditions (Poethke *et al*., 2016; Varpe, 2017). By spreading risk, the population avoided wipeout by starvation in anomalous late-bloom years while still attempting to maximize fitness (fecundity and survival) in “ordinary” years; note that BH is not a population-level strategy but the outcome of variability in the strategies of individual copepods. Two types of BH arose in the model simulations, conservative and diversified (Liu *et al*., 2019). Predicted optimal exit from diapause was relatively late, on day 110, compared to most of the RY simulations although still well in advance of the peak of the spring bloom in all years (conservative BH). High genetic and phenotypic diversity is expected in copepod populations (Bucklin and Kocher, 1996; Kann and Wishner, 1996; Unal and Bucklin, 2010). In contrast to the RY simulations, predicted optimal PV was high (SDPV = 20 days), thereby enabling a range of exit dates over several months that provides flexibility to respond to anomalous conditions (diversified BH). This optimal PV was however intermediate within the range investigated indicating that while too little restricts the response to anomalous conditions, too much PV can be wasteful if many of the resulting phenotypes result in trophic mismatch (Bandara *et al*., 2021a). When phenotypic variance was excluded from the IA simulations, the modeled copepods had to compromise further by exiting diapause on day 120 (sensitivity analysis with SDPV = 0).

Demographic time-series sampling of *C. finmarchicus* at Station Mike in 1997 showed a major cohort of adult females arriving in surface waters on day 95, more than a month ahead of the observed spring chlorophyll peak on day 140 (Heath *et al*., 2000a). A good analog for these data in our analysis is year 4 in the 10-year sequence (Fig. 4): the spring bloom peak occurs on day 140. Predicted diapause exit date on day 100 in the corresponding RY simulation is close to the observations while exit occurred slightly later, on days 110 in the IA simulations. While these predictions for diapause exit at Station Mike are broadly in line with observations, it should be noted that close agreement is not necessarily expected for several reasons. Our environmental forcing was model-derived and is itself subject to uncertainties (food and temperature fields were from the NEMO-MEDUSA model, Yool *et al*., 2013). The observed time-series at Mike is the outcome of a series of different water parcels passing through a fixed point as determined by the 3D flow field of the surrounding area (Heath *et al*., 2000b; Samuelsen *et al*., 2009; Huse *et al*., 2018), confounding time-series analysis (Hind *et al*., 2000). It should also be remembered that optimality studies are not necessarily about accurately reproducing all of the diversity and complexity of nature. Our simulations took many decades to reach convergence using an end-on-end sequence of years, whereas real ecosystems are in a continuous state of flux. The aim of optimality studies it is rather to investigate optimal trait combinations, which confer maximum fitness under specified conditions. Much can be learned about the relative importance of different constraints that influence the evolution of life history strategies in animals (Parker and Maynard Smith, 1990) and thereby how to parameterize copepod life history and phenology in biogeochemical models.

Losses due to predation dominated mortality of the modeled copepods with only a minor contribution from starvation in most years. Predation was specified as an increasing trend during the growing season in the model. *Calanus* spp. are the primary prey of the herring in the Norwegian Sea (Dalpadado *et al*., 2000; Gislason and Astthorsson, 2002) and the feeding intensity of these and other fish builds up during the growing season (Dalpadado *et al*., 2000; Kaartvedt, 2000; Gislason and Astthorsson, 2002) enhanced by increasing daylength that boosts visual predation (Kaartvedt, 2000; Varpe *et al*., 2007; Varpe and Fiksen, 2010). Model results indicate that early exit from diapause is a favorable life history strategy when predation is low early in the year because eggs spawned pre-bloom, and subsequent naupliar and copepodite stages, are less susceptible to predation losses (Varpe *et al*., 2007). As a sensitivity test, the seasonal trend of increasing predation was replaced with fixed (seasonally invariant) predation rates (Lynch *et al*., 1998; Miller *et al*., 1998b; Maps *et al*., 2010). Top-down pressure is then relatively higher early in the year which led to a delay in predicted diapause exit of 20 days (from 110 to 130) in the IA simulations, and by up to 40 days in the RY simulations, as the copepods minimized the risk of being eaten prior to peak food availability. In contrast, day of diapause exit showed relatively little sensitivity to directly increasing predation pressure by 50 or 100% while maintaining the seasonal trend. Our results emphasize the importance of representing the seasonality of predation pressure in order to reliably simulate the population dynamics of *C. finmarchicus* in ocean biogeochemical models.

Diel vertical migration (DVM) offers an escape mechanism by which copepods can offset visual predation losses (Cisewski *et al*., 2021), as shown by several modeling studies (Fiksen and Giske, 1995; Fiksen and Carlotti, 1998; Bandara *et al*., 2018, 2019). Incorporating DVM would be an interesting future addition to our model, requiring an extra trait to represent the trade-off between predator avoidance and diminishing access to food resources. It should be noted, however, that a significant fraction of the predation experienced by *C. finmarchicus* may accrue from non-visual predators including chaetognaths, ctenophores and carnivorous copepods whose abundance often correlates with their prey, but which also varies seasonally and among years (Sullivan and Meise, 1996; Dale *et al*., 2001; Ohman *et al*., 2008; Yaragina *et al*., 2022). Other future developments of the model include the addition of capital production and a one-year life cycle where *C. finmarchicus* transitions directly from CV to adults without entering diapause (Broms *et al*., 2009). The latter could be incorporated into the model as an additional trait (diapause or no diapause) or by assuming a fixed fraction of the population enters diapause (Speirs *et al*., 2006). Representing and simulating individual copepods with the two life cycles alongside each other is a major task and is beyond the scope of the current work.

Populations of *C. finmarchicus* will likely experience phenological shifts in phytoplankton bloom timing that are expected in response to climate warming (Henson *et al*., 2018) and in coastal areas due to changes in optical properties of the water column (Opdal *et al*., 2019). It is essential that the cues of diapause exit are identified if we are to understand and predict how these populations will respond to variability of this kind. A critical unresolved issue is whether copepods can sense surface-originating irradiance while in the latter stages of diapause. If they are unable to do so (Østvedt, 1955; Hind *et al*., 2000), they most likely rely on endogenous cues and/or bet-hedging strategies. Endogenous mechanisms such as exhaustion or selective catabolism of lipid reserves (Irigoien, 2004; Johnson *et al*., 2008; Pond, 2012; Biggs *et al*., 2020; Bandara *et al*., 2021a) or internal biological clocks (Häfker *et al*., 2018) can generate a wide range of diapause exit dates but can be unreliable as many of them may bear little correspondence to bloom timing (Miller *et al*., 1991; Bandara *et al*., 2021a). Phenotypic plasticity provides an alternative means of diversified bet-hedging, as indicated by the results presented herein. Separately, an interesting two-stage mechanism for exit from diapause in *C. finmarchicus* was hypothesized by Melle *et al*. (2003). Triggered by an endogenous cue, the first stage involves ascending to relatively shallow depths at which light can be perceived and used to cue the second step, ascent to sunlit surface waters. This final ascent could correspond directly with bloom timing if the animals can detect changes in the light spectrum resulting from absorption by phytoplankton pigments or can directly sense sinking algae (Melle *et al*., 2003).

Incorporating the SLP and its response to changing climate into Earth System Models is currently at the cutting-edge of oceanographic research. What can we recommend regarding the parameterization of diapause exit in these models? Our results indicate that optimal exit is generally timed to coincide with the onset of the spring bloom. In turn, bloom timing occurs later with increasing latitude in the North Atlantic (Henson *et al*., 2009) due to delayed stratification of the water column. One could simply use latitude to scale diapause exit timing in an ESM but it is arguably better to use responsive metrics that correlate with latitude such as net heat flux, mixed layer depth shoaling or average mixed layer photosynthetically active radiation (Cole *et al*., 2015). Photoperiod is another timing option but is questionable because, after the spring equinox, a given daylength occurs earlier at high latitudes than at lower latitudes (an inverse trend). Diversified bet-hedging could be included by enacting diapause exit of the zooplankton community in stages over a period of weeks or months. If one accepts the hypothesis of Melle *et al*. (2003), which is as yet unsupported, diapause exit could instead be linked directly to the onset of the spring bloom, e.g. using a threshold phytoplankton concentration.

There is a lot more than parameterizing diapause exit when it comes to incorporating the SLP into ESMs and the model of *C. finmarchicus* employed here, LILICOP_1.0 (Anderson *et al*., 2022), provides a good starting point. As well as including a lipid reservoir, it incorporates the latest C/N stoichiometry (necessary because biogeochemical models invariably have a nutrient element, usually N, as their base currency) that includes explicit metabolic terms that are temperature-sensitive, along with standard equations for grazing, growth, etc. The use of an individual-based approach is, however, precluded in ESMs where, due to computational constraints, copepods are normally represented as bulk state variables in units of biomass per volume (e.g. mmol N m^−3^). This distinction in model framework introduces several issues that complicate application of LILICOP to ESMs. For example, diapause entry in LILICOP occurs when individual copepods reach a fixed individual lipid content. In the case of bulk models, this information is unavailable and it could take instead take place when, for example, phytoplankton levels drop below a threshold concentration (Hind *et al*., 2000) or when seasonal water column stratification breaks down. Furthermore, to reign in model complexity and computational cost, copepods are often grouped together with other mesozooplankton that may not share the same phenology and seasonal diapause behavior. Simplified approaches tailored to this reduced complexity will therefore be required.

## CONCLUSION

Our study indicates that the optimal phenology of diapause in *C. finmarchicus* is strongly dependent on the variability of environmental forcing. In predictable (stable) environments, optimal exit from diapause is tied to the early onset of the spring phytoplankton bloom and populations successfully propagated over multiple generations without the need for phenotypic variance. Early exit from diapause is a favorable strategy in environments with seasonally increasing predation pressure, enhancing survival of eggs and early developmental stages. Copepods are in reality exposed to unpredictable fluctuations in environment such as interannual variability in bloom timing and predation in which case they have to compromise, giving rise to reduced fitness, when it comes to optimizing the phenology of their life history. Bet-hedging strategies, including phenotypic variance, enable populations to deal with anomalous events associated with highly variable (unpredictable) environmental forcing. Representing the SLP in the Earth System Models used to project climate change will benefit from an improved understanding of the cues of diapause exit and careful consideration of how to represent both these and bet-hedging strategies in an environment characterized by strong spatio-temporal variability.

## Supplementary Material

Anderson_supplemantary240424_fbae028

## Data Availability

The LILICOP_2.0 model is coded in R as stand-alone files (main R file, “LILICOP_2.0.R”, along with input files for model parameters and forcing), with no dependency on R libraries. The model files are freely available for online download at the Zenodo repository; the citation is Anderson, T.R. (2024). LILICOP_2.0: model of LIpids in the LIfe cycle of a high latitude COPepod, version 2.0. Zenodo, doi: 10.5281/zenodo.11036475.
