# Supplementary material for: Optimal phenology of life history events in Calanus finmarchicus: exit from diapause in relation to interannual variation in spring bloom timing and predation
Source: J Plankton Res. 2024 Jun 7;46(4):439–51. doi: 10.1093/plankt/fbae028 (PMC11290252; doi:10.1093/plankt/fbae028)
Supplement: Anderson_supplemantary240424_fbae028 [file anderson_supplemantary240424_fbae028.docx]

**Optimal phenology of life history events in *Calanus finmarchicus*: exit from diapause in relation to interannual variation in spring bloom timing and predation: Supplementary Information**

Journal of Plankton Research

Thomas Anderson, Dag. O. Hessen, Wendy C. Gentleman, Andrew Yool, Daniel J. Mayor

**Supplementary Appendix 1: Summary of trait convergence results**

A summary of the simulations undertaken (interannual IA and repeat-year RY years RY1 to RY10), showing trait convergence results, is presented in Tables S1.1 (optimal diapause exit) and S1.2 (optimal phenotypic variance, PV, expressed as standard deviation, SDPV). For example, “10×110” indicates 10 simulations (ensemble size), each of which resulted in predicted optimal exit on day 110. Trait outcomes in the default simulations are highlighted in blue and results which differed from the default results in other simulations are highlighted in red. The different simulations are: default, predation mortality (m) fixed (no interannual variability), predation mortality +50%, +100% (maintaining the seasonal trend), and no phenotypic variance.

Table S1.1. Predicted optimal diapause exit day of year for the simulations undertaken

Default m fixed m +50% m +100% no PV

IA 10×110 10×130 1×100, 9×110 10×110 10×120

RY1 5×100 5×120 5×100 5×100 1×100

RY2 5×150 5×160 5×150 4×150, 1×160 1×150

RY3 5×120 5×130 5×120 5×120 1×120

RY4 5×100 5×100 5×100 5×100 1×100

RY5 5×100 5×110 5×100 5×100 1×100

RY6 5×100 5×110 5×100 5×100 1×100

RY7 5×80 5×120 5×80 5×80 1×80

RY8 5×100 5×120 5×100 5×100 1×100

RY9 5×90 5×110 5×90 5×90 1×90

RY10 5×100 5×130 4×100, 1×110 2×100, 3×110 1×100

Table S1.2. Predicted optimal phenotypic variance (SDPV, days)

Default m fixed m +50% m +100% no PV

IA 10×20 10×0 1×10, 9×20 5×20, 4×30, (10×0)

1×40

RY1 5×0 5×0 5×0 5×0 (1×0)

RY2 5×0 5×0 5×0 5×0 (1×0)

RY3 5×0 5×0 5×0 5×0 (1×0)

RY4 5×0 5×0 5×0 5×0 (1×0)

RY5 5×0 5×0 5×0 5×0 (1×0)

RY6 5×0 5×0 5×0 5×0 (1×0)

RY7 5×0 5×0 5×0 5×0 (1×0)

RY8 5×0 5×0 5×0 5×0 (1×0)

RY9 5×0 5×0 5×0 5×0 (1×0)

RY10 5×0 5×0 5×0 5×0 (1×0)

The main differences in predicted trait convergence from default are in the fixed predation mortality simulations which showed (i) later optimal diapause exit in all simulations (IA and RY) except for RY4, and (ii) SDPV was zero in the IA simulation, unlike SDPV = 20 days using default parameters settings.

Repeatability (same convergence result) was demonstrated in most simulation settings except at high predation mortality where there were minor variations that do not affect our overall conclusions. Population size is in effect smaller in the face of high mortality, meaning that random selection of individuals during the predation mortality process can have a greater stochastic bearing on emergent outcomes.

The simulation results where phenotypic variance was excluded are bracketed because optimisation was only for optimal diapause exit day. In case of the RY simulations, predicted emergent outcomes were expected to match those of the corresponding default simulations (which had optimal SDPV = 0) and so only a single simulation (ensemble size of 1) was undertaken for each scenario for confirmatory purposes.

**Supplementary Appendix 2. Number of generations to trait convergence**

The number of generations required to achieve convergence of optimal diapause exit and phenotypic variance expressed as standard deviation (SDPV) in the interannual (IA) simulations (10-member ensemble) is shown in Fig. S2-1. Optimal diapause exit day usually converged by generation 150, i.e., requiring only fifteen passes of the 10-year interannual sequence. In contrast, SDPV took considerably longer to converge, requiring the simulation of 200-800 generations, showing marked difference between the different model runs.


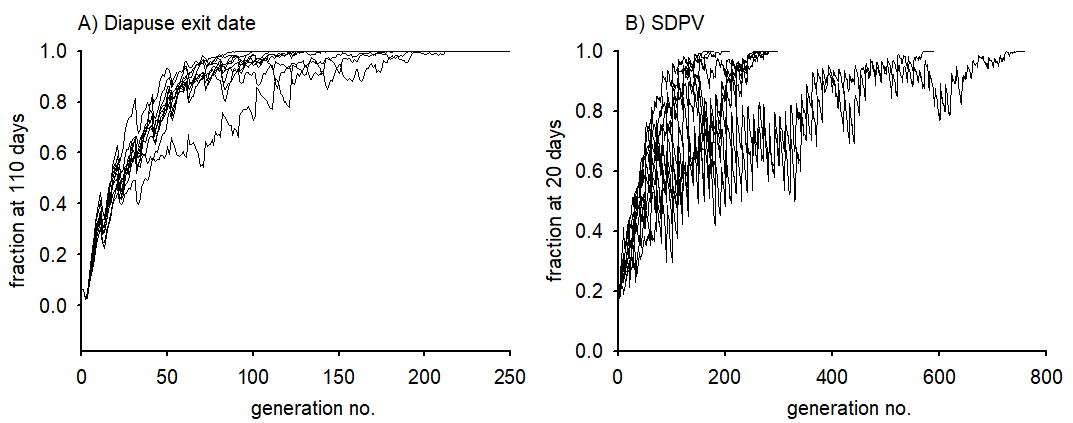


Fig. S2-1. Time taken to trait convergence (number of generations) for all IA simulations in the 10-member ensemble, A) optimal diapause exit day, B) SDPV. Note difference in x-axis scaling between the two panels.

A typical IA simulation takes ~2 hours on a standard desktop PC, noting that our chosen sub- population size is 408,000 for the first 20 generation and 100,000 thereafter (the population was uniformly, i.e., maintaining trait frequencies and egg spawn dates, reduced at the juncture). The number of generations required to achieve convergence increases if a larger sub-population size is selected or the resolution of trait bins is increased, as well as depending on model parameter settings such as predator mortality (which affects the effective population size).

**Supplementary Appendix 3: Comparison of results of the 10-member ensemble of the interannual simulation: survivorship and fecundity**

Our standard simulation with interannual forcing (10 years, 2000-2009) used a sub-population size of 408,000 for the first 20 generations and 100,000 thereafter until trait convergence. Parameter values were set to default, including predator mortality probabilities of 0.03, 0.002 and 0.02 d^-1^ during development to CV, diapause and as adults. A 10-member ensemble was carried out. Results from the first simulation are presented in the main text. Here, we compare survivorship and fecundity across the ensemble to demonstrate reproducibility of the results, noting the stochastic nature of predator mortality. Predicted trait values at convergence were the same in all simulations: exit date from diapause of day 110 and SDPV = 20 days (Supplementary Appendix 1).

Survivorship (adults egg^-1^, i.e., the number of adults produced per spawned egg) and fecundity (eggs adult^-1^, i.e., number of eggs produced per adult emerging post-diapause) are the two key factors that determine propagation of the population from one generation to the next (see Supplementary Appendix 6 for further details). Optimised trait values operate to ensure successful propagation, which is the product of survivorship and fecundity, throughout the interannual sequence. The predicted variability in survivorship (adults egg^-1^) for a given year within the ensemble (Fig. S3-1A) was small (mean ± SD): yr 1 (0.023 ± 0.00035), yr 2 (0.0068 ± 0.00024), yr 3 (0.024 ± 0.00086), yr 4 (0.017 ± 0.00064), yr 5 (0.014 ± 0.00020), yr 6 (0.027 ± 0.00075), yr 7 (0.011 ± 0.00043), yr 8 (0.011 ± 0.00032), yr 9 (0.0094 ± 0.00028), yr 10 (0.018 ± 0.00056).


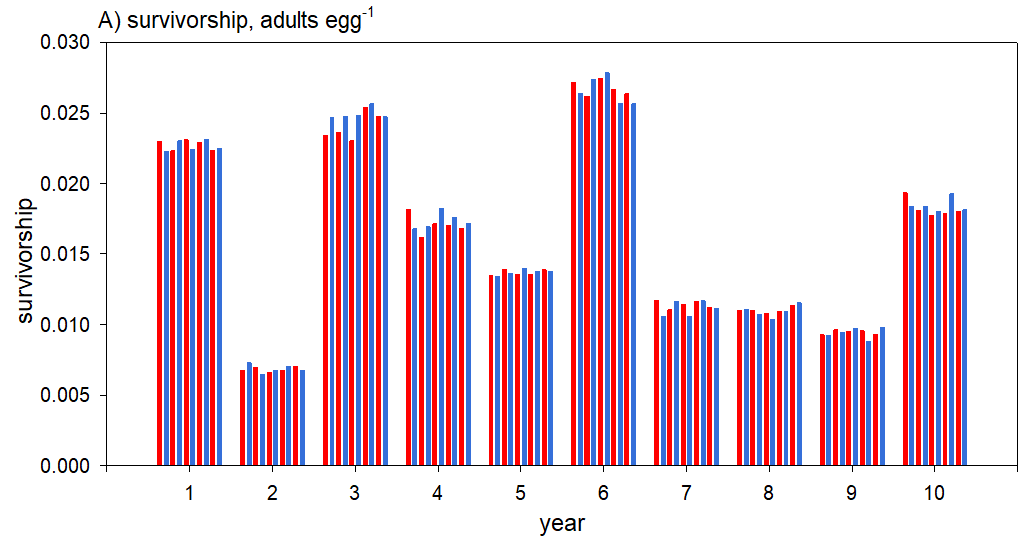


Fig. S3-1A. Predicted survivorship (adults egg^-1^) for the 10-member ensemble, ordered (x-axis) by the 10 years in the interannual sequence; red and blue colouration for visual separation of the bars.

Predicted fecundity (eggs adult^-1^); Fig. S3-1B) likewise varied little between different members of the ensemble: yr 1 (851 ± 11), yr 2 (115 ± 8), yr 3 (689 ± 26), yr 4 (804 ± 10), yr 5 (761 ± 19), yr 6 (842 ± 19), yr 7 (709 ± 9), yr 8 (728 ± 16), yr 9 (725 ± 21), yr 10 (705 ± 19).


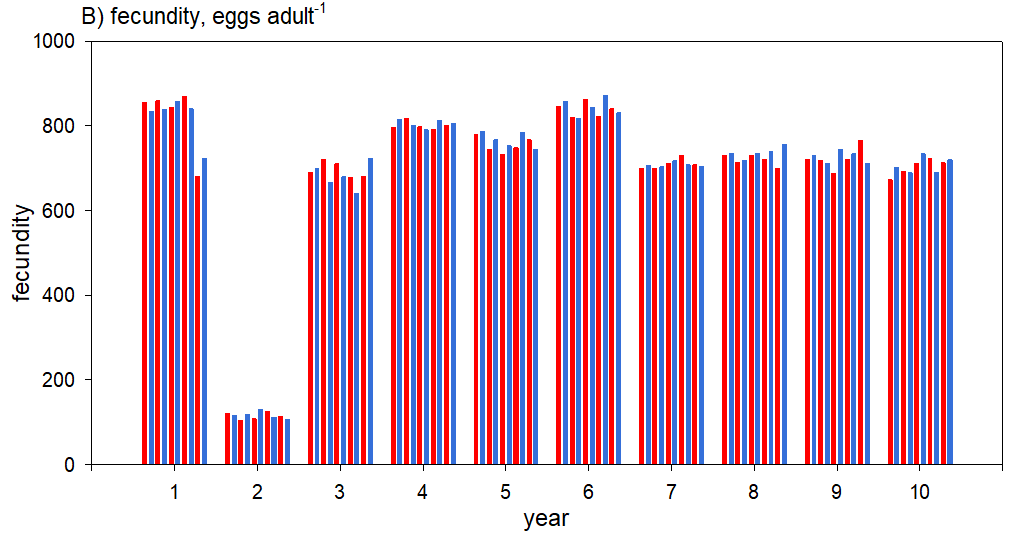


Fig. S3-1B. Predicted fecundity (eggs adult^-1^) for the 10-member ensemble, ordered (x-axis) by the 10 years in the interannual sequence.

**Supplementary Appendix 4: Relationships between exit date from diapause and food, temperature**

The relationship between predicted optimal diapause exit day of year and seasonal cycles of food for the RY simulations are shown in Fig. 4 (main text). We found a strong positive correlation, close to the 1:1 line, when exit day is plotted against day of bloom onset defined as the point in time at which food first reaches 2 mmol C m^-3^ (Fig. S4-1):


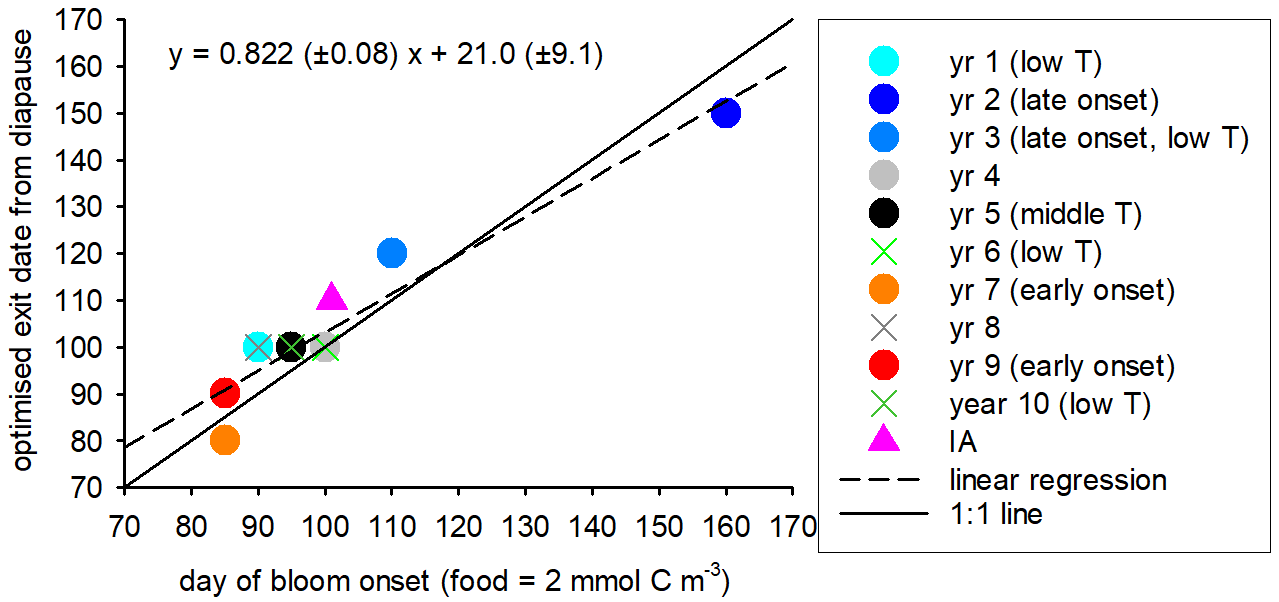


Fig. S4-1. Relationship between optimised diapause exit date and bloom onset (defined as the day of year when food first reaches 2.0 mmol C m^-3^). Also shown is the result of the IA simulation, using x = 101 which is the average of the RY runs (not included in the regression). Colour-coding as in Fig. 3 (main text). 1:1 line also shown.


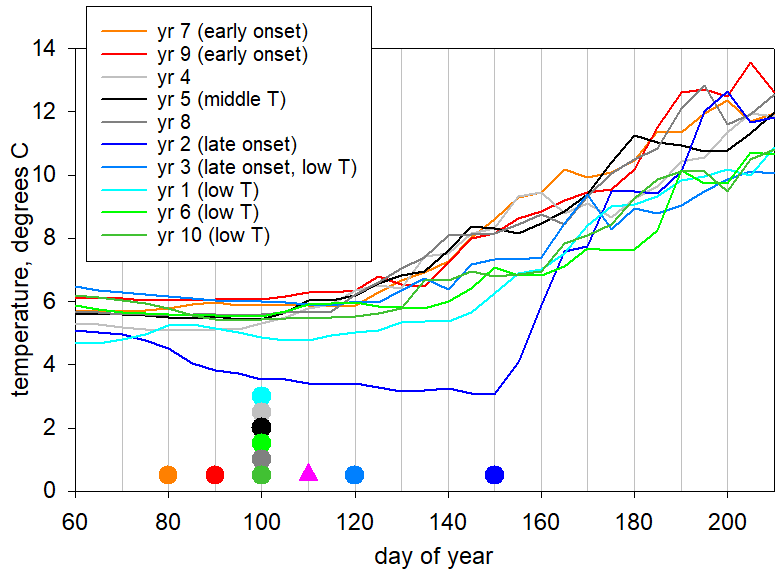


Fig. S4-2. Predicted distributions optimal exit date from diapause for the repeat-year simulations (spots) and the interannual simulation (pink triangle), plotted against the seasonal cycles of sea surface temperature. The colour scheme highlights different years according to bloom timing and temperature (T): early bloom (years 7, 9; orange, red), late bloom (years 2, 3; bloom, pale blue), low-temperature (years 3, 1, 6, 10; pale blue, cyan, green, olive).

There is no obvious relationship between predicted diapause exit date and temperature (Fig. S4-2). There may be a negative relationship when these variables are potted against each other (here using the surface temperature at day 100 as the x variable; Fig. S4-3), but it is less convincing than the relationship shown in Fig. S4-1. In general, one might expect a negative correlation between sea surface temperature and the timing of bloom onset because the former is an indicator of the extent of vertical mixing in the water column in the water column (inverse of stratification).


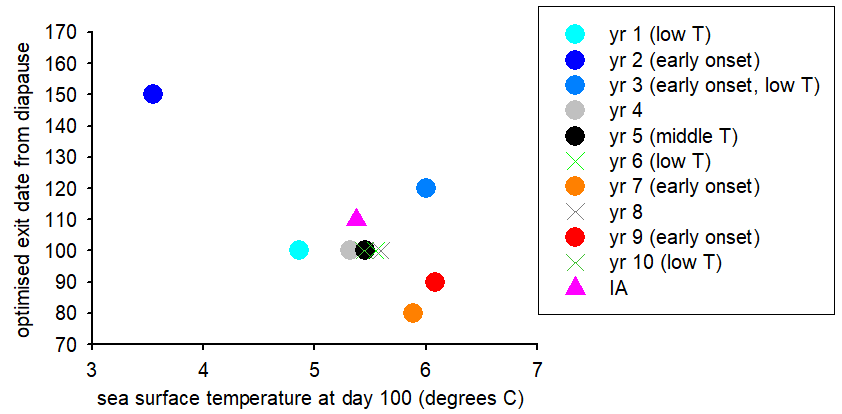


Fig. S4-3. Relationship between optimised diapause exit date and see surface temperature at day 100. Also shown is the result of the IA simulation, using x = 5.37 which is the average of the RY runs. Colour-coding as in Fig. 3 (main text).

**Supplementary Appendix 5: Fecundity and egg fate for the interannual and repeat-year simulations**

The predicted seasonal progression of average egg production per adult female in years 2 and 9 were shown in Fig. 5 (main text), highlighting the interaction with food and temperature (predation pressure) and showing egg fate (starvation, predation, becoming adults). Here, we show similar plots for all ten years in the interannual sequence (Figure S5-1).

**
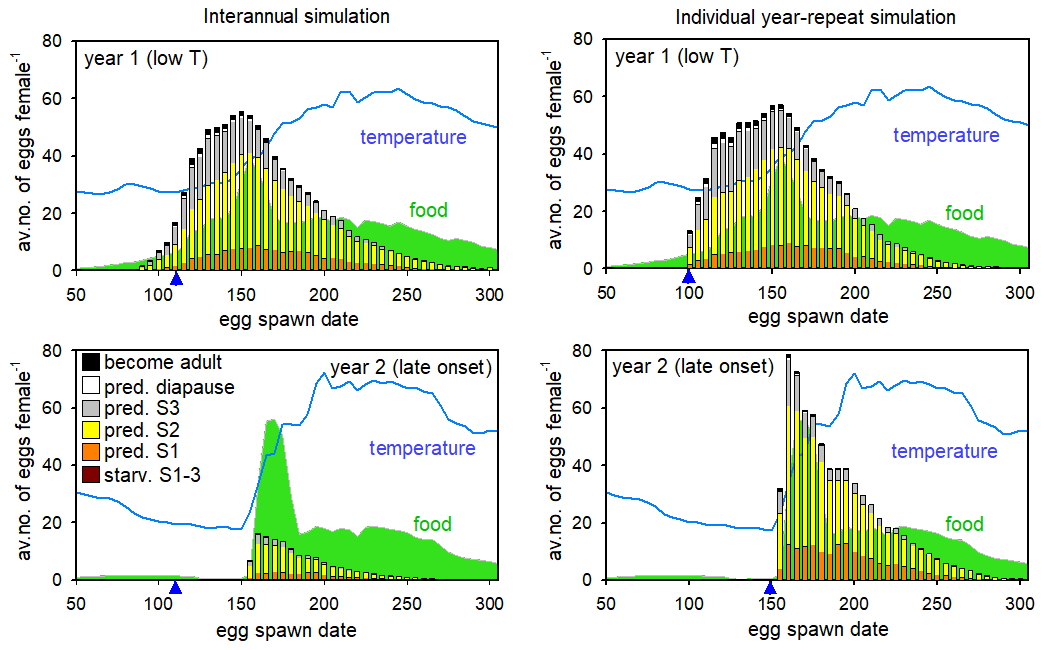
**

**
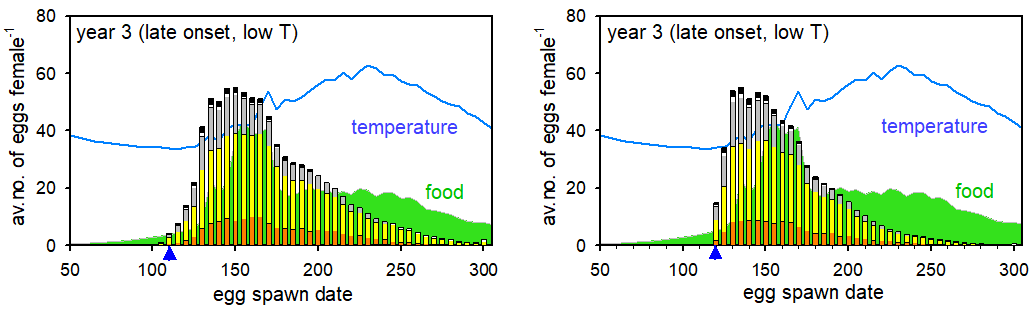
**

**
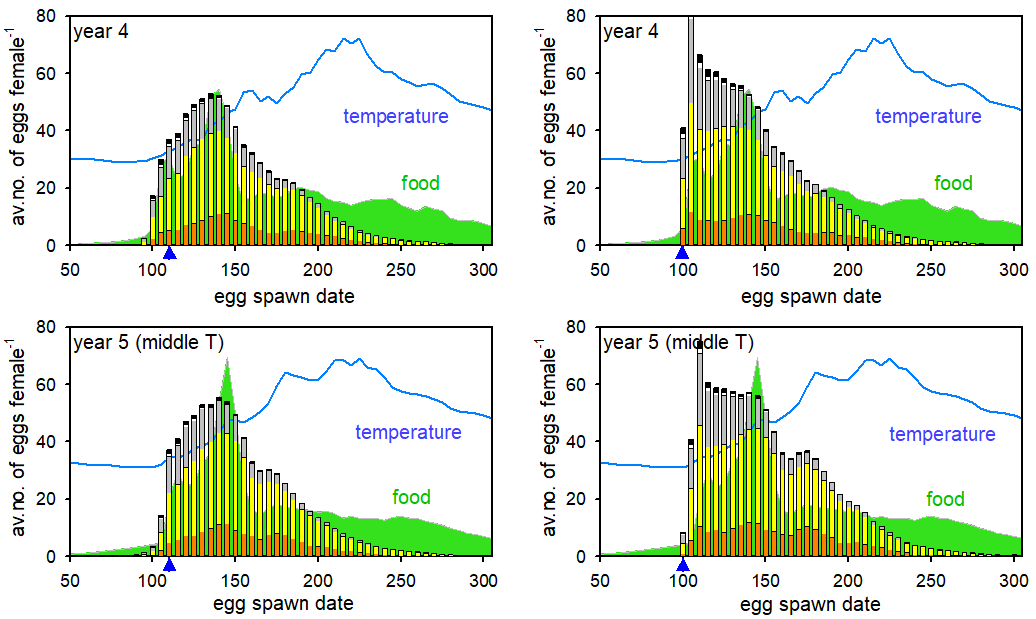
**

**
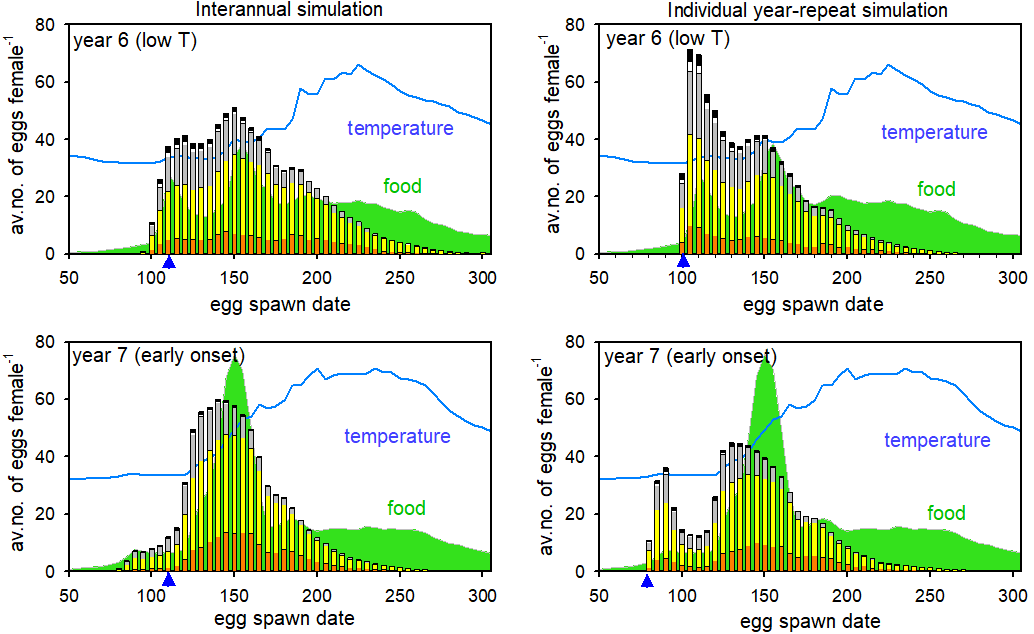
**

**
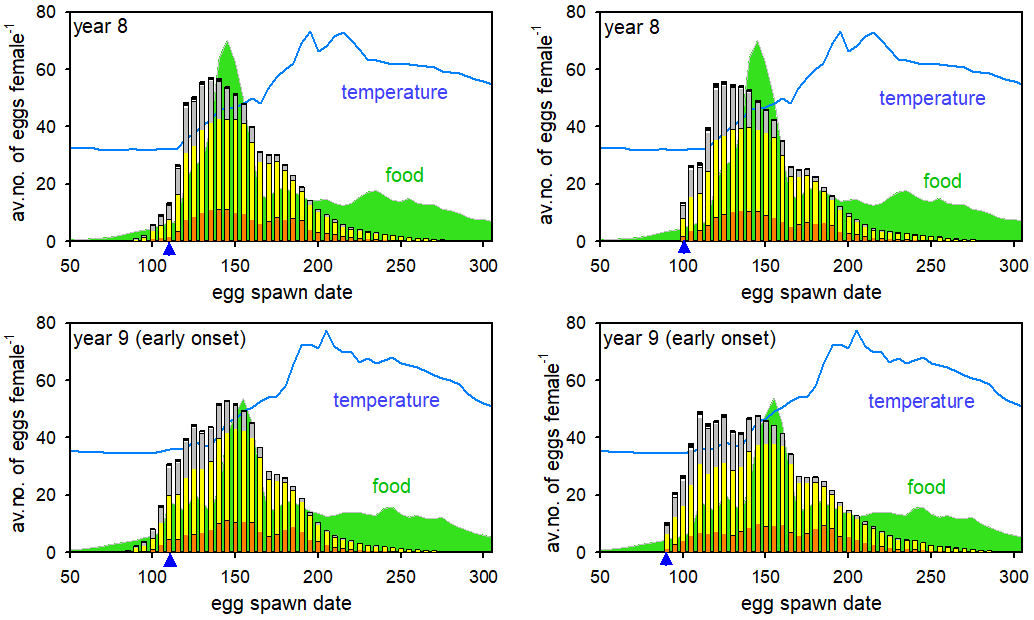
**

**
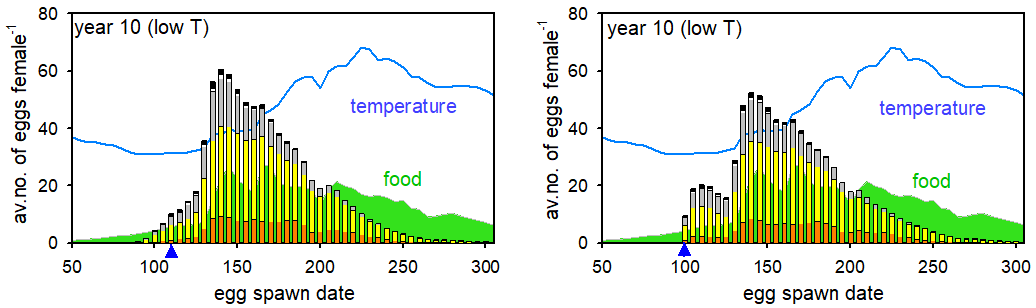
**

Fig. S5-1. Predicted seasonal progression of average egg production per adult female the interannual and repeat-year simulations (blue triangles indicate optimal exit date from diapause) for all ten years (years 2 and 9 shown in Fig. 5 of main text). Bar colouration shows the ultimate fate of eggs spawned on different dates: starvation during development (Stages 1-3), predation Stage 1 (eggs and non-feeding nauplii), predation Stage 2 (feeding and growth without lipid deposition), predation Stage 3 (feeding and growth with lipid deposition), predation during diapause, and survivorship through to becoming adults. Seasonal cycles of food (green shading) and temperature are also shown (scaling as in Fig. 3 main text).

The seasonal progression of total eggs spawned that subsequently successfully become adults in the interannual and repeat-year simulations for years 2 and 9 was shown in Fig. 6 (main text). Here, we show results for all ten years (Fig. S5-2).

**
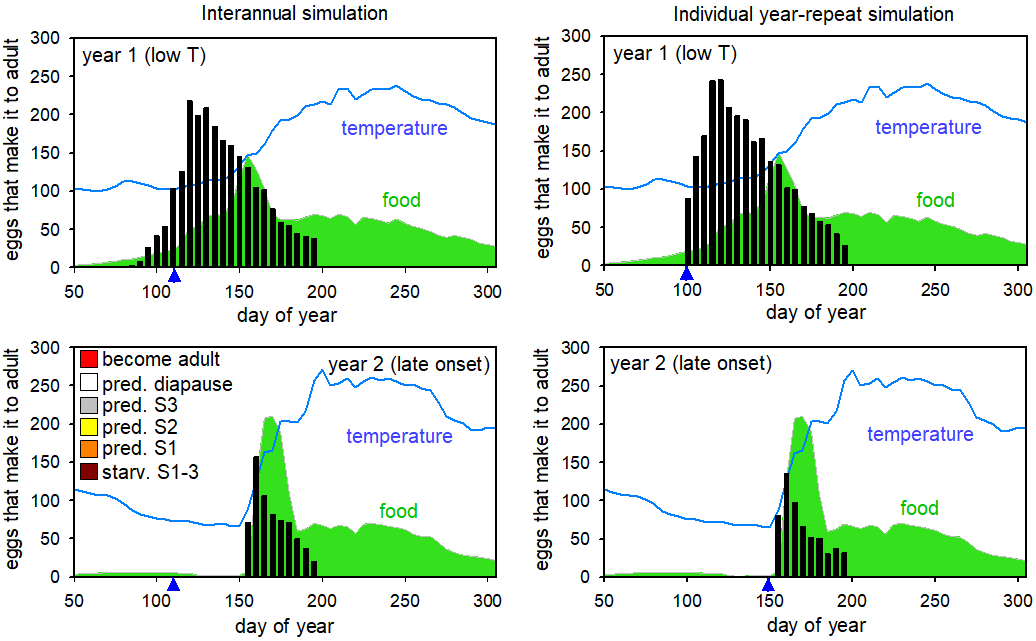
**

**
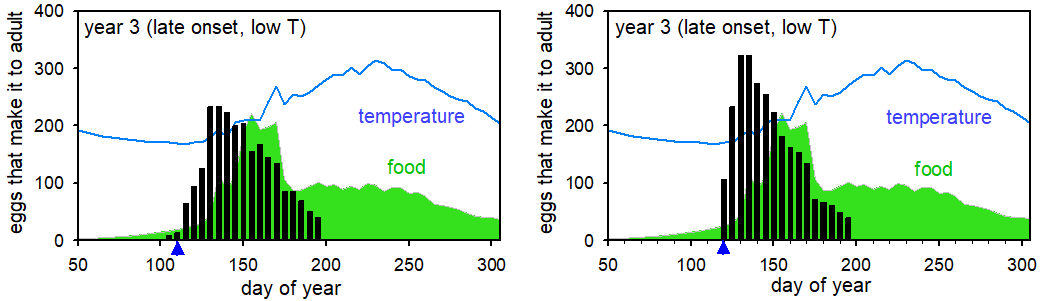
**

**
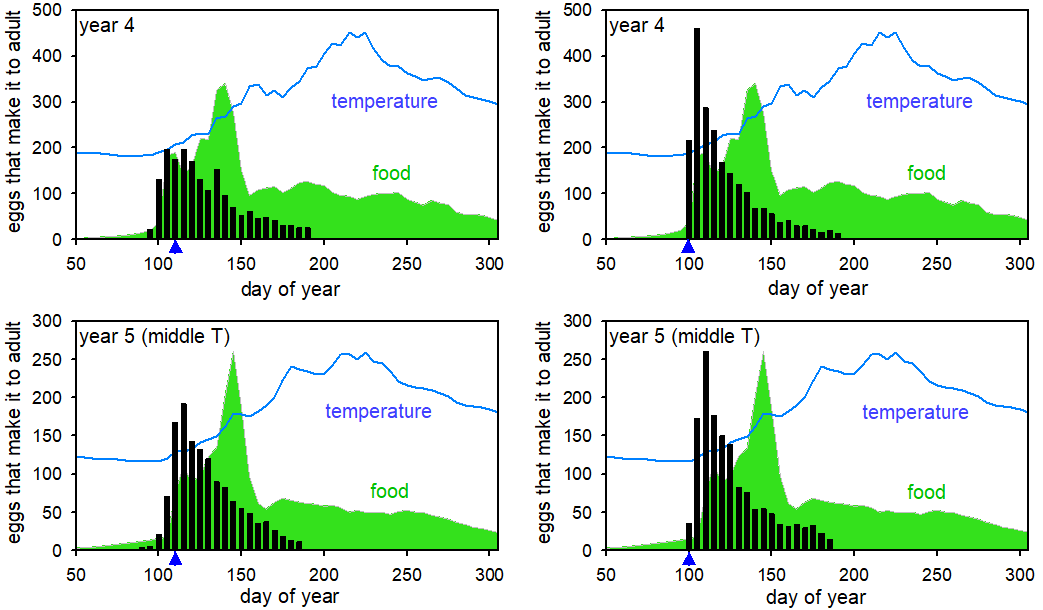
**

**
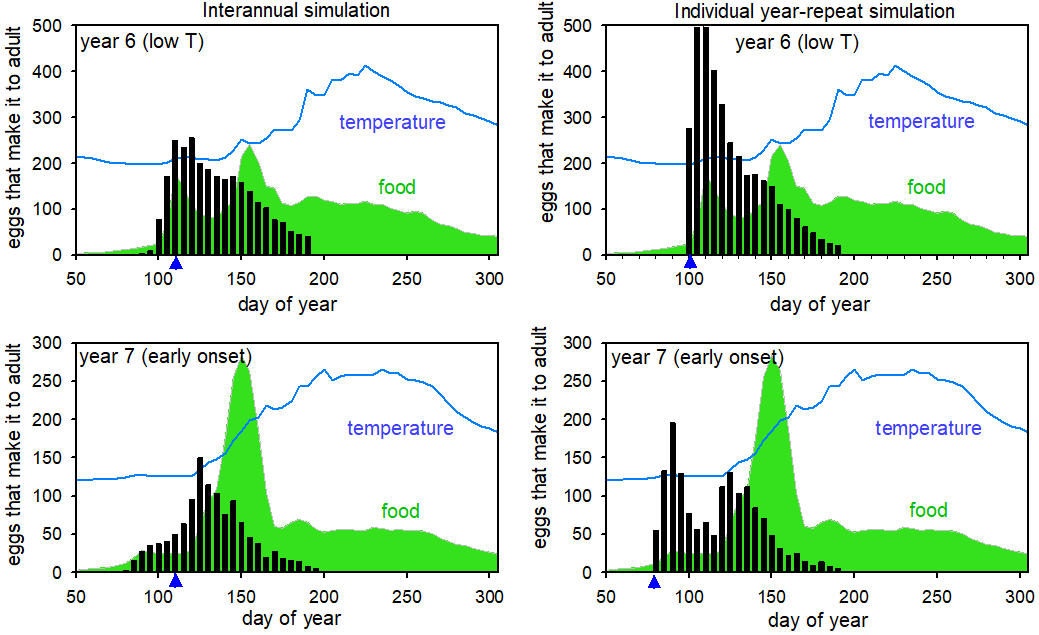
**

**
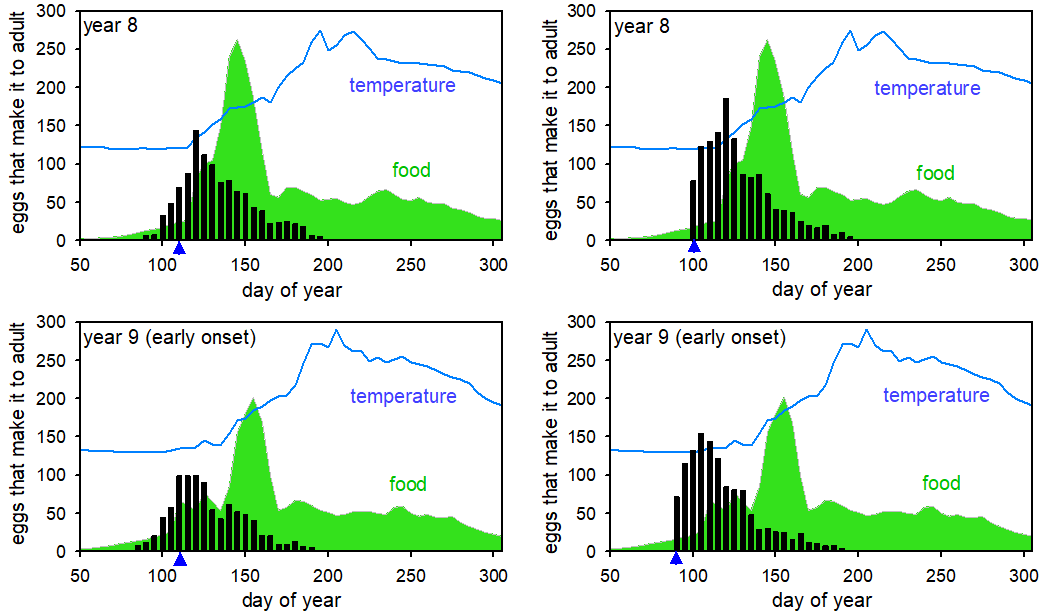
**

**
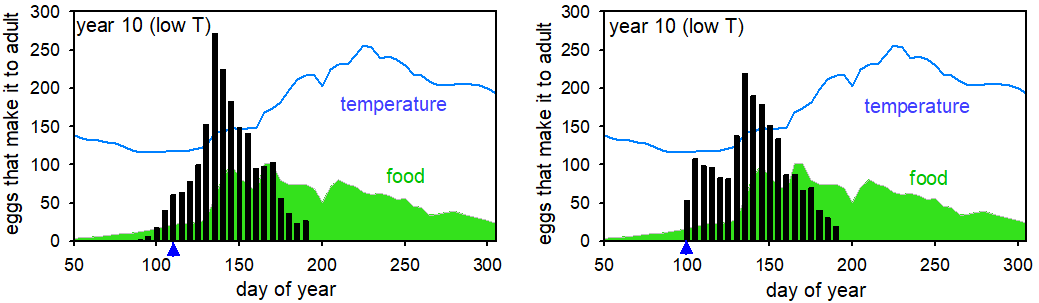
**

Fig. S5-2. Predicted seasonal progression of total eggs spawned that subsequently successfully reach CVI (become adults) in the interannual and repeat-year simulations (blue triangles indicate optimal exit date from diapause) for all ten years. Seasonal cycles of food (green shading) and temperature are also shown (scaling as in Fig. 3 main text).

**Supplementary Appendix 6: Population propagation**

The success with which the sub-population of copepods is propagated from one generation to the next is the product of fecundity (eggs female^-1^) and the fraction of eggs which complete the life cycle and emerge as adults (adult females egg^-1^ in the second year of the life-cycle). These metrics are shown for the IA and RY simulations in Fig. S6-1.


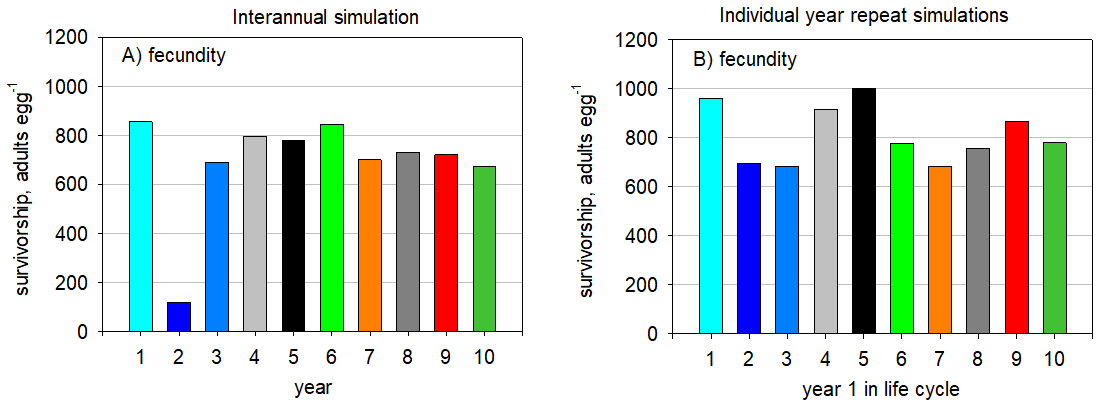


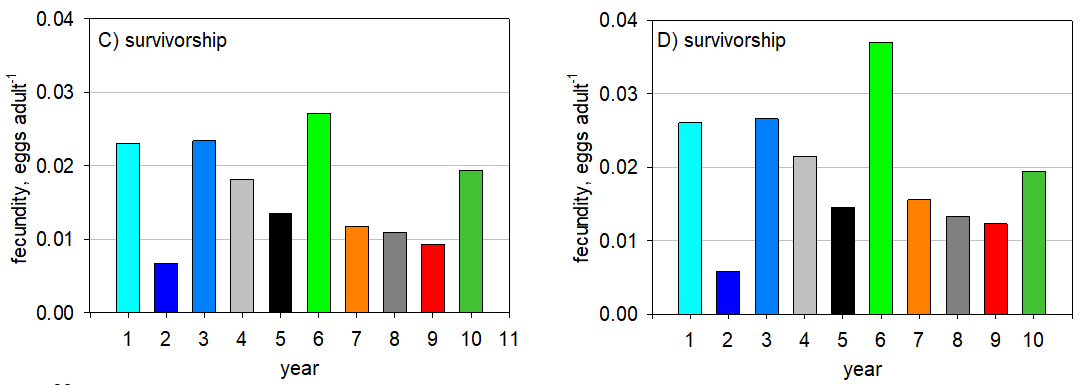


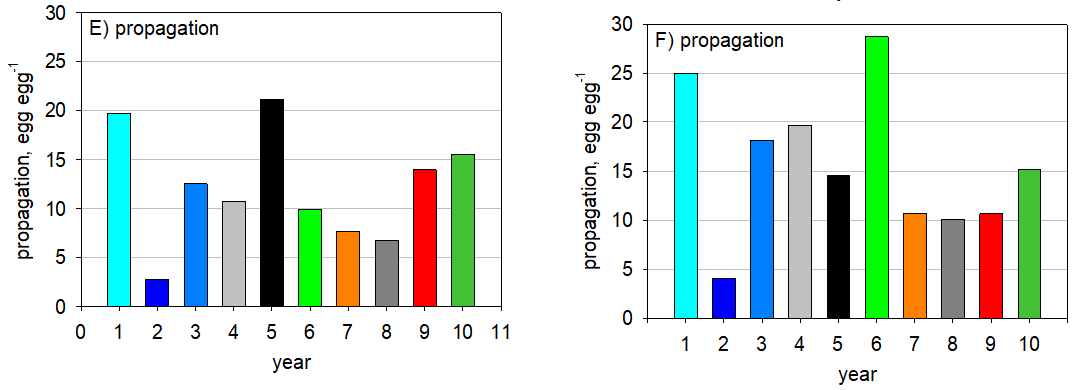


Fig. S6-1. Average fecundity (adults egg^-1^; panels A, B), survivorship (eggs adult^-1^; panels C, D) and propagation (of the sub-population from one generation to the next; panels E, F) for the IA and RY simulations. Colouration as in Fig. 3 (main text): early onset bloom years (7, 9; orange, red), late onset years (2, 3; blue, pale blue), low temperature years (1, 3, 6, 10; cyan, pale blue, green, olive).

Predicted fecundity is high in all years in the IA simulation except year 2 when it was restricted by the mass starvation of adults (Fig. S6-1A). Less than 3% of eggs reach the adult phase in all years (Fig. S6-1C, D). Survivorship is highest in years where predation pressure is lowest during development to CV (Fig. 6-1C, D), i.e., the colder years (1, 3, 6, 10). Year 2 is an exception because, although it is also a cold year, exit from diapause did not take place until late in the season (day 150) by which time predation pressure was increasing rapidly.

Propagation of the sub-population is highest in year pairings 3-4 (first and second years of the life-cycle), 6-7 and 10-1 due to the high survivorship from egg to adult in these instances. It is lowest across years 1-2 because of low fecundity. Propagation is generally higher in the RY simulations because exit from diapause is optimal with respect to the timing of the spring phytoplankton bloom, improving survivorship by reducing starvation (Fig. 6-1E, F).

**Supplementary Appendix 7: Derivation of Gaussian distributions**

Gaussian distributions were calculated using the standard normal distribution formula:

$f\left( x \right)=\frac{1}{\sigma\sqrt{2\pi}}exp(-0.5\left( \frac{x-\mu}{\sigma} \right)^{2})$ (Eq. S7.1)

where μ and σ are the mean and standard deviation, respectively.

For practical reasons (long tails may cause stability issues in the model code) we truncated the distribution with -1 ≤ x ≤ 1. Predicted f(x) for μ = 0 and σ = 0.2, 0.4, 0.6, 0.8 is then (Fig. S7-1):


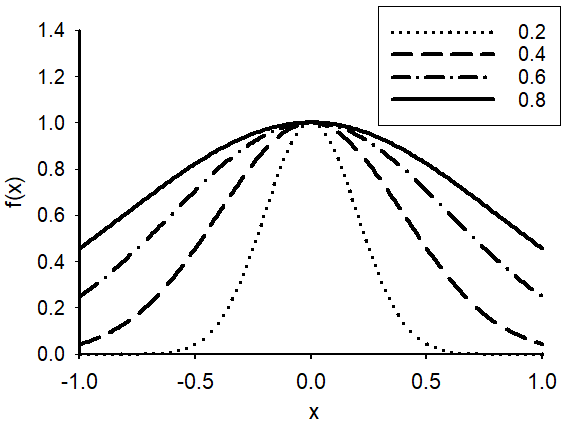


Fig. S7-1. Truncated (in x) Gaussian distributions for σ = 0.2, 0.4, 0.6, 0.8.

Normalising to give the same area under each curve, and reconstituting the x-axis from -50 to +50 then results in the Gaussian distributions used in the model (Fig. 2 is repeated here):


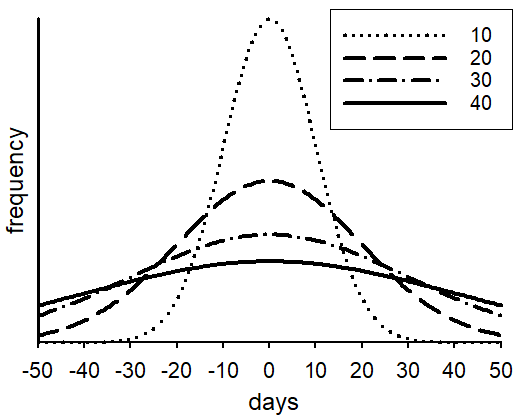


Fig. 2 (repeated from main text). Frequency distributions for phenotypic variance of diapause exit with standard deviation, SDPV = 10, 20, 30, 40 days.
